# RETRACTION: Effect of Endoscopic Retrograde Appendicitis Therapy on Surgical Site Wound Infection and Hospital Stay in Patients With Acute Appendicitis: A Meta‐Analysis

**DOI:** 10.1111/iwj.70313

**Published:** 2025-03-06

## Abstract

**RETRACTION:** WangY. , MaL. , LuX. , and YanX. , “Effect of Endoscopic Retrograde Appendicitis Therapy on Surgical Site Wound Infection and Hospital Stay in Patients With Acute Appendicitis: A Meta‐Analysis,” International Wound Journal 20, no. 10 (2023): 4281–4290, 10.1111/iwj.14330.37500538 PMC10681414

The above article, published online on 27 July 2023, in Wiley Online Library (http://onlinelibrary.wiley.com/), has been retracted by agreement between the journal Editor in Chief, Professor Keith Harding; and John Wiley & Sons Ltd. Following an investigation by the publisher, all parties have concluded that this article was accepted solely on the basis of a compromised peer review process. The editors have therefore decided to retract the article. The authors did not respond to our notice regarding the retraction.

**RETRACTION:** Y. Wang , L. Ma , X. Lu , and X. Yan , “Effect of Endoscopic Retrograde Appendicitis Therapy on Surgical Site Wound Infection and Hospital Stay in Patients With Acute Appendicitis: A Meta‐Analysis,” International Wound Journal 20, no. 10 (2023): 4281–4290, 10.1111/iwj.14330.37500538 PMC10681414

The above article, published online on 27 July 2023, in Wiley Online Library (http://onlinelibrary.wiley.com/), has been retracted by agreement between the journal Editor in Chief, Professor Keith Harding; and John Wiley & Sons Ltd. Following an investigation by the publisher, all parties have concluded that this article was accepted solely on the basis of a compromised peer review process. The editors have therefore decided to retract the article. The authors did not respond to our notice regarding the retraction.

